# Developing a Taxonomy of Dark Triad Triggers at Work – A Grounded Theory Study Protocol

**DOI:** 10.3389/fpsyg.2017.00293

**Published:** 2017-03-07

**Authors:** Annika Nübold, Josef Bader, Nera Bozin, Romil Depala, Helena Eidast, Elisabeth A. Johannessen, Gerhard Prinz

**Affiliations:** ^1^Department of Work and Social Psychology, Maastricht UniversityMaastricht, Netherlands; ^2^Faculty of Psychology and Educational Sciences, University of CoimbraCoimbra, Portugal; ^3^Department of Psychology, University of LjubljanaLjubljana, Slovenia; ^4^Department of Experimental Psychology, University of OxfordOxford, UK; ^5^Independent ResearcherTallinn, Estonia; ^6^Department of Psychology, University of WinchesterWinchester, UK; ^7^Department of Basic Psychological Research and Research Methods, University of ViennaVienna, Austria

**Keywords:** dark triad, personality states, workplace triggers, taxonomy, grounded theory

## Abstract

In past years, research and corporate scandals have evidenced the destructive effects of the dark triad at work, consisting of narcissism (extreme self-centeredness), psychopathy (lack of empathy and remorse) and Machiavellianism (a sense of duplicity and manipulativeness). The dark triad dimensions have typically been conceptualized as stable personality traits, ignoring the accumulating evidence that momentary personality expressions – personality *states* – may change due to the characteristics of the situation. The present research protocol describes a qualitative study that aims to identify triggers of dark triad *states* at work by following a grounded theory approach using semi-structured interviews. By building a comprehensive categorization of dark triad triggers at work scholars may study these triggers in a parsimonious and structured way and organizations may derive more effective interventions to buffer or prevent the detrimental effects of dark personality at work.

## Introduction

The study of dark personality and its impact in the workplace has gained increasing attention in the past years (Spain et al., 2014). Dark personality traits are defined as characteristics that reflect a motivation to elevate the self and harm others (Paulhus and Williams, 2002). Amongst other conceptualizations (e.g., Hogan and Hogan, 2001) the dark triad consisting of narcissism, psychopathy, and Machiavellianism (Paulhus and Williams, 2002) represents the most popular operationalization of dark personality at work (Spain et al., 2014).

*Narcissism* is characterized by feelings of grandiosity, entitlement, dominance, and superiority (Spain et al., 2014). People who show this trait tend to be charming or pleasant in the short term while in the long run presenting difficulty in maintaining successful interpersonal relationships, lacking trust and care for others (Morf and Rhodewalt, 2001). *Psychopathy* involves feelings of impulsivity, thrill-seeking, low empathy and anxiety. Those that present a psychopathic trait seek immediate gratification of their needs, lack guilt and conscience, being less prone to experience embarrassment and failing to learn from punishment for misdeeds (Hare, 1985). *Machiavellianism* is associated with cynicism, low affect, an unconventional view of morality and a focus exclusively on personal goals (Christie and Geis, 1970). Thus, those who exemplify this trait tend to be exceedingly willing to manipulate others and take a certain pleasure in successfully deceiving them (Jones and Paulhus, 2014).

Importantly, despite similarities and overlap, the dark triad is not identical with clinically relevant personality disorders nor does it reflect simply extreme forms of normal personality traits (Harms et al., 2014). Both research and past corporate scandals have evidenced the dark triad’s effects on counterproductive work behaviors (e.g., O’Boyle et al., 2012; Spain et al., 2014) and a variety of other destructive outcomes, such as heightened competitiveness, dysfunctional job crafting, and corruption (Carter et al., 2014; Roczniewska and Bakker, 2016; Zhao et al., 2016).

To date, research has mainly focused on the detrimental *outcomes* of dark personality. Although initial efforts have been made in past years to discover the psychological underpinnings of the dark triad (Paulhus and Williams, 2002), research has failed to address the role of situational factors in eliciting momentary expressions of dark personality characteristics. To date, the dark triad has exclusively been conceptualized and investigated in its trait-like form, ignoring evidence for the malleability and short-term fluctuation of personality states, the expression of one’s personality in a specific moment (e.g., Fleeson, 2001). Due to the predominant view of the dark triad as stable traits, situational cues eliciting dark triad behavior have not been of concern so far. Attempts to identify the roots of dark triad personality have thus focused on very broad, generic explanations, such as evolutionary (e.g., Jonason et al., 2009), behavioral genetic (e.g., Vernon et al., 2008), socio-ecological (Jonason et al., 2016), neuro-biological (Jonason and Jackson, 2016), and motivational (Harms et al., 2014; Jonason and Ferrell, 2016) foundations.

**FIGURE 1:**
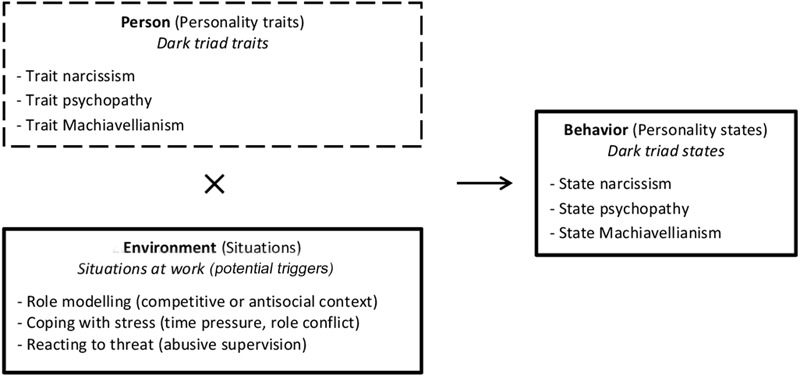
**Conceptual model following Lewin’s equation for behavior (Lewin, 1936): *B = f(P, E)***.

In our study we apply a more dynamic approach to dark personality. Drawing on interactionist models of personality, such as the cognitive affective personality system (CAPS; Mischel and Shoda, 1995, 1998) or whole trait theory (Fleeson and Jayawickreme, 2015) that build upon Lewin’s (1936) equation for predicting behavioral reactions [*B* = *f*(*P* ×*E*)], we assume that stable personality traits (*P*) interact with environmental characteristics (*E*) to produce a specific behavioral response (*B*), or in other words, a specific personality state (see **Figure 1** for our conceptual model; please note that the listed situations at work merely represent examples of potential triggers and don’t reflect empirical findings). Mischel and Shoda (1995, 1998) called this complex interplay *if-then contingencies* describing the idea that specific situational cues make people reliably react in a specific way, based on their personality traits. Empirical studies (e.g., Judge et al., 2013) and entire special issues (Beckmann and Wood, 2016) have evidenced that the behavioral expressions (i.e., personality states) in relation to changing situations present a potentially predictable reflection of personality (Mischel and Shoda, 1995, 1998).

Despite the growing evidence that personality expressions are dependent on situational cues, research on the triggering function of job characteristics is still in its infancy and has only focused on adaptive personality states so far (Judge et al., 2013; Dóci and Hofmans, 2015). The usual approach for adaptive personality states to conceptualize situations mainly as opportunities to express one’s personality (Ten Berge and De Raad, 1999) may, however, be problematic for the concept of the dark triad. Whereas some situations may allow for or even encourage the expression of dark personality characteristics because they may be functional in that moment (e.g., being self-aggrandizing in a selection interview), other situations may rather trigger dark personality states because specific needs and motives are not fulfilled (e.g., the need for power). Thus, although “a psychology of situations has begun to take shape” (Funder, 2016, p. 203), a comprehensive taxonomy of situational *triggers* of *specific* personality characteristics, including the dark triad, has yet to be established.

In this study, we aim to identify the underlying *situational* antecedents in the work environment (E) that lead to within-person variation in momentary dark triad expressions, i.e., in dark triad personality states (B), that is, *state* narcissism, *state* psychopathy and *state* Machiavellianism, resulting in a comprehensive taxonomy of triggers. We use a grounded theory approach (Strauss and Corbin, 1998; Corbin and Strauss, 2008) as this qualitative methodology is particularly suited for complex social processes about which little is known yet (Glaser and Strauss, 1967; Willig, 2009). Identifying potential triggers of dark personality expressions at work and building taxonomy of these triggers is important in several ways. On a theoretical level, a more comprehensive understanding of the triggers of dark personality states and their potential interconnections is crucial in order to acquire knowledge on the nomological network and common mechanisms that may be associated with specific groups or categories of triggers. Moreover, identifying triggers of dark personality expressions at work is practically important, as organizations strongly benefit from detailed knowledge on situations that may “make people snap.” Organizations and Human Resource (HR) professionals will be able to design evidence-based interventions that help prevent employees from expressing their dark tendencies which may bring harm to organizations and their members over the short and long term. These actions may not only include job design and selective placement, but also training and coaching interventions that sensitize employees for potentially dangerous situations and helping them to either themselves better manage and regulate their dark impulses or increase their ability to cope with others’ dark behaviors, enabling better relationships and work ethics within their organizations.

With the present study, we contribute to the literature in three important ways: Firstly, identifying triggers of *dark* personality expressions at work adds to the evolving personality state literature by broadening the domain of situational predictors and allowing us to better understand the complex interplay between person and situation characteristics (P × E) jointly leading to the expression of personality states (B) in a specific situation. Secondly, our taxonomy may be used to further advance research in the field of dark personality at work by stimulating the creation of instruments such as questionnaires or situational judgment tests that enable scholars to study these triggers in quantitative studies in a more standardized and parsimonious way. In addition, our taxonomy may further add to the study of long-term personality development, helping to further explore how individuals’ personality turns dark over time by investigating how short-term dynamics add up in a longitudinal fashion (Hogan et al., 1994). Finally, editors of top tier journals (e.g., Suddaby, 2006; Bansal and Corley, 2011) explicitly acknowledge the value of grounded theory and call for more qualitative research in the organizational literature. By following this call we promote a methodology that is particularly useful for examining situated processes like employee interactions in complex organizational settings (Locke, 2001). Qualitative research complements the many quantitative studies in the organizational literature by offering the reader a close-up of the phenomenon being studied and providing the opportunity to raise new research questions, revealing “deeper insights into management, organizations, and society, which are critical to understanding and potentially shaping our world” (Bansal and Corley, 2011, p. 235).

In sum, our research questions are as follows:

(1) Which situational characteristics at work trigger expressions of state narcissism?
(2) Which situational characteristics at work trigger expressions of state psychopathy?
(3) Which situational characteristics at work trigger expressions of state Machiavellianism?

## Materials and Equipment

### Semi-structured In-depth Interviews

In order to gather information on momentary experiences of dark triad states and their eliciting factors at work, we will conduct semi-structured in-depth interviews with employees. Semi-structured interviews enable the interviewer to flexibly adapt and add further questions depending on the answers given by the interviewee allowing for more in-depth explanations of how the person experienced the situation, thereby increasing the validity of the interview (McLeod, 2014).

Interviews will be conducted with jobholders of all seniority levels reporting about their own experiences (i.e., self-reports) or about the behavior of someone else (i.e., observer-reports), to account for different perspectives and to create a multifaceted view on the phenomenon of interest (Glaser and Strauss, 1967; Bluhm et al., 2011). As we expect it to be more difficult to obtain answers from participants about their own dark behaviors due to the possibility of socially desirable responding, we will first invite participants to talk about their own behaviors before also offering them the option of reporting the behaviors of a significant other (e.g., a colleague, supervisor or subordinate). Although self-reports are particularly valuable as they can target internal emotions and cognitions, it is important to note that research has evidenced that dark triad characteristics (e.g., features of psychopathy) can also be reliably and validly detected by lay raters, particularly if they involve interpersonal behaviors (Fowler et al., 2009). Therefore, employees reporting on their impressions and observations of a relevant situation involving *another* person are valuable sources of information as well (Fowler et al., 2009).

Although it has also been recommended to apply several methods of data collection (Bluhm et al., 2011), conducting observations and analyzing existing written materials will not be the focus of our research. As our study’s purpose is to identify the (to date unknown) triggering factors of dark triad states, it will neither be possible to reasonably plan specific observation periods nor to conduct observations spanning many hours or even days hoping for a potential triggering situation to occur.

The actual interview will consist of three main parts:

(1) An open and generic question about the interviewees’ experiences at work and their job in general in order to allow interviewees to get comfortable with reporting about their experiences while we introduce them to the topic.
(2) The second part will include questions about experiences of specific dark triad expressions at work. Descriptions of these dark personality expressions will be based on items from validated instruments, such as the Mach IV (Christie and Geis, 1970), the Self-Report Psychopathy Scale III (SRP-III; Hare, 1985), and the Narcissistic Personality Inventory (NPI; Raskin and Hall, 1979). These descriptions will ensure a common understanding of the focal behaviors and will stimulate interviewees to reflect on situations in which they have shown these behaviors themselves (or observed them in another person). A sample question for state Machiavellianism would be: “When thinking about your work experiences in the past 12 months – can you think about a specific situation in which you have done certain things to get someone to do what you want?”. In cases where participants experience difficulties in answering the question, a prompt could be: “For instance, have you said something particularly to someone to get what you want?”. Interviewees will then be asked to describe a *specific situation* in which they expressed this behavior in more detail, for example in terms of where the situation took place, the specific individuals involved in the interaction, and the specific actions.
(3) The third step will focus on the aim of the interviews, the potential triggering situations of dark triad expressions at work. Accordingly, interviewees will be asked their opinion on what they believe may have *evoked* their specific behavior, that is, the preceding situation and the specific trigger of that behavior. Interviewees will also be asked about the broader context of the situation (e.g., if there have been prior incidents with someone), as the meaning of situational triggers may change from one context to another. Participants will be asked to describe this triggering situation in as much detail as possible. As suggested by previous research (Judge et al., 2013; Jonason and Ferrell, 2016; Jonason et al., 2016), we will ask participants reporting their own experiences for both, more objective environmental events (i.e., something someone did or said) and internal events (e.g., subjective feelings, motives, or cognitions), as both events may serve as antecedents to dark triad personality states. Participants reporting the behaviors of someone else will only be asked about the more objective features of the situation and will not be asked to speculate about the potential thoughts and feelings of the target person. As noted by Funder (2016) with reference to Reis (2008), situations are always filtered through the perception of the individual who experiences them, but should nonetheless be conceptualized separately from individual construal. Likewise, Rauthmann et al. (2015) emphasize in the so called processing principle that people’s experiences are based upon objective environmental events which are explicitly and/or implicitly processed by individuals, thereby providing situations with meaning. The perspective that both internal and external events may be meaningful as triggers of dark personality states is in line with a post-positivist perspective and the aim to come closer to a singular truth while acknowledging that evidence is not confined to what can be physically observed and that subjective influences exist.

## Methods

### Design

In the present study we use a grounded theory approach to answer our research questions. Grounded theory captures the complexity of social processes like no other methodology, it reveals content that is highly embedded in practice, and gives researchers the possibility to describe a phenomenon in great detail (Martin and Turner, 1986; Strauss and Corbin, 1998). Most importantly, it supports theorizing in “new” areas of research (Strauss and Corbin, 1998; Birks and Mills, 2015) and allows researchers to revise the direction and framework of research in real time as soon as new information and findings emerge. Our research questions are particularly appropriate for a grounded theory approach as there is a lack of research on dark personality states and therefore also a lack of essential information on their triggers.

There are multiple philosophies regarding grounded theory methodology (e.g., Glaser, 1978; Strauss and Corbin, 1998; Corbin and Strauss, 2008; Charmaz, 2014). In the present study, we follow the epistemological approach of *postpositivism* which assumes that there is one truth that can be discovered, but acknowledges that individuals’ perceptions are influenced by the context and that information gathered in the research process is not a neutral reflection of the truth. Thus, we approach grounded theory with the understanding that reality exists and that objectivity can be reached by discovering an emergent theory that represents this reality as accurately as possible. In line with the post-positivist approach, we follow Strauss and Corbin’s (1998) assumption that a theory is *discovered* in the data instead of being fully constructed.

Although cross-cultural research has many benefits, conducting interviews in two different languages (English and German) may be an area of concern in qualitative studies (Squires, 2009; Nurjannah et al., 2014). As recommended for multilingual research projects (Van Nes et al., 2010), we aim to make use of the original language for as long as possible. Specifically, interviews that will be conducted in English will be transcribed and coded in English while the interviews conducted in German will be transcribed and coded in German. Only after the coding procedure, we will translate the codes and the respective text passages and sample quotes derived from the German interviews into English. In order to ensure equivalence of meaning of these translations, we will follow the translation back-translation procedure by Brislin (1970), while making use of a translator moderator (Van Nes et al., 2010). The translator moderator will be the first author who will conduct the interviews in German and at the same time is highly proficient in English. Translating the codes instead of the original transcripts ensures the authenticity of the data and quality of analysis by minimizing potential misinterpretation and loss of participants’ intended meanings (Larkin et al., 2007) while at the same time being more economic and feasible (Chen and Boore, 2009).

### Participants

In order to identify triggers of dark triad expressions at work, we will approach jobholders that are either willing to report on their own behavior at work or on behavioral observations of someone else, for example a colleague, their supervisor or a subordinate. In addition, we will approach subject matter experts, such as HR consultants, who may be able to report on dark triad expressions of clients and potential triggering situations based on their work with organizations. As the dark triad (Paulhus and Williams, 2002) refers to *subclinical* or *everyday versions* of maladaptive personality, in contrast to clinically relevant disorders, dark triad characteristics may be well represented in normal populations. Thus, our target sample will consist of regular employees and professionals. Potential participants will initially be approached via the interviewers’ networks (e.g., via professional business and employment-oriented social networking service).

In order to achieve high heterogeneity of data sources (i.e., a maximum amount of variance in the target behaviors and situational triggers), we plan to approach male and female employees of different ages from a wide variety of branches, jobs, positions, and hierarchy levels. Through this approach, we also aim to identify more severe situations (that may be needed to trigger individuals with low levels in the dark triad) as well as less critical situations (that may function as triggers for those with high dark triad levels). We will not determine the number of interviews (our sample size) *a priori* (Eisenhardt and Graebner, 2007), but will continue to collect data until a *theoretical saturation point* has been reached and no new relevant categories of triggers emerge (Glaser and Strauss, 1967). As qualitative research handles non-numerical information and because the right sample size depends on a number of factors, such as the variety and content of answers and the scope of the study, power calculations that are appropriate in quantitative research are not applicable here (Morse, 2000; Leung, 2015). Nonetheless, scholars have for example suggested 20–30 interviews for grounded theory (Creswell, 1998), a sample size that has also been confirmed in grounded theory studies conducted in an organizational context (Seivwright and Unsworth, 2016; Wilhelmy et al., 2016). Additionally, we will document the specific steps of the theoretical sampling process to make the choice of our eventual final sample size as transparent as possible (Nelson, 2016).

### Procedure

**FIGURE 2:**
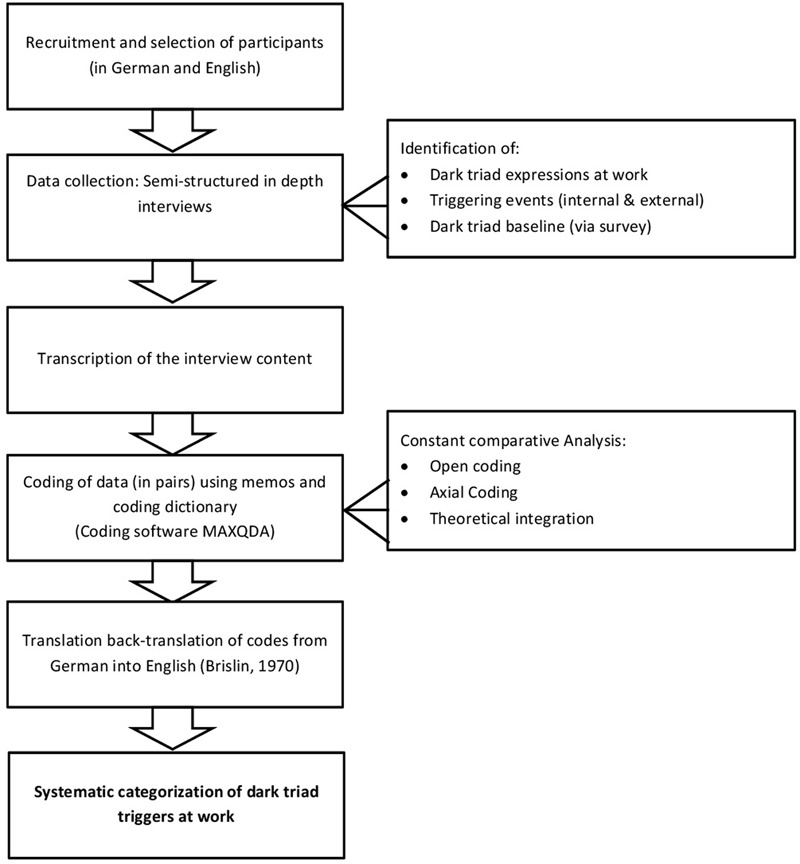
**Model of the step-by-step procedure following a grounded theory approach**.

A model of the procedural steps in our study is depicted in **Figure 2**.

Interviews will be conducted in English and German. All interviews will be audio recorded after obtaining permission from participants to tape-record the session. Interviews will take place face-to-face at the facilities of the university the respective interviewer is affiliated with or via telephone or videoconference calls (e.g., Skype). Telephone and videophone calls have been found to be a solid substitute for face-to-face interviews, especially in semi-structured interviews, while at the same time allowing for more efficient data collection (Sturges and Hanrahan, 2004; Berg, 2007; Sullivan, 2012).

At the beginning of each in-depth interview, participants will be informed about the purpose and general context of the study in order to ensure transparency and allow for proper consideration of participation (e.g., Wilhelmy et al., 2016). Further, individuals will be ensured of the anonymity and confidentiality of their answers and the right to withdraw from the study at any point. In order to limit recall bias, participants will be instructed to respond to our interview questions based on their work experiences within the past 12 months. At the end of each interview, participants will be encouraged to complete a short questionnaire of the dark triad traits, the SD3 (Jones and Paulhus, 2014), in order to capture their baseline level of these characteristics. The assessment of their baseline will allow for a more detailed understanding of the distribution and level of dark personality characteristics in our sample and will allow us to link this information with the descriptions of the triggering situations, enabling us to control for possible moderating effects (P × E). Research has frequently documented that personality characteristics (P), such as neuroticism, moderate the perception of and reaction to daily experiences (E) (e.g., stressful events; Bolger and Zuckerman, 1995; Suls and Martin, 2005) and systematically influence the level and variability of personality states (B) (Fleeson and Jayawickreme, 2015). Likewise, individuals with different levels of dark triad traits may perceive and react to situations differently (either in terms of the quality and character or the severity of situational cues). In order to prevent sensitization effects, we decided to administer the SD3 (Jones and Paulhus, 2014) after rather than before the interview.

Participants will be asked to sign the informed consent form and give general demographic related information. Although it is possible that participants will find some content of the interview upsetting or disturbing, this is very unlikely (Corbin and Morse, 2003). Reporting on sensitive topics can even benefit participants as it gives them an opportunity to be heard and to express their thoughts and feelings (Corbin and Morse, 2003). Participants will also have the opportunity to request that their interviews are not used in our study and the option to withdraw at any stage. Finally, individuals will be asked if they can recommend further potential interview candidates who would be suited and willing to participate in the study while bringing a benefit to our project (i.e., snowballing procedure).

Information gathered throughout the interviews will be used to develop new and more detailed questions to be added to the interview guide as well as to adjust our sampling strategy by, for example, targeting additional branches (Glaser and Strauss, 1967; Eisenhardt and Graebner, 2007). Through this procedure we will be able to further verify ideas that emerged from previous interviews and to ensure that we gather a rich and comprehensive view on the situations and identify interrelations between triggers, common trends, as well as their respective validity and importance (Glaser and Strauss, 1967).

### Proposed Analysis

As recommended by Corbin and Strauss (2008), data will be analyzed and discussed by multiple researchers (i.e., the authors). All interviews will be coded by pairs of two researchers to ensure multiple perspectives on the data (Corbin and Strauss, 2008) while minimizing personal bias and increasing reliability of our coding procedure. For the coding of the transcripts we will use the coding software (MAXQDA, 1995–2017). The transcriptions will be coded in three different stages – open coding, axial coding and theoretical integration (Corbin and Strauss, 2008). Coding is a procedure through which researchers create meaningful labels for sections of texts. Using coding to make sense of the data is not a step-by-step procedure, but rather a very flexible, iterative process (Rich, 2012). The different stages do not have to be followed in a strict manner but the data may take the analysis back and forth until *theoretical saturation* is reached and no new coding categories emerge (Pandit, 1996; Strauss and Corbin, 1998). In order to facilitate the coding procedure, we will make use of a so-called coding dictionary, a document including the evolving system of categories that will be continually modified through constant comparative analysis of existing and newly evolving codes (Kreiner et al., 2009). In order to maintain a high theoretical sensitivity, it is essential not to emerge oneself in the existing literature, as this may lead to biased results; researcher may unconsciously search for specific information to “fit” previous findings and fail to identify other important concepts inherent in the data (Strauss and Corbin, 1998).

The first stage of coding is open coding where the data is fractured or broken down into discrete parts which are closely examined and compared and then provided with codes which at a later stage can be grouped into categories (Glaser and Strauss, 1967). In general, codes can be given to the data word-by-word, phrase-by-phrase, sentence-by-sentence, line-by-line, or paragraph-by-paragraph (Strauss and Corbin, 1998). We will use line-by-line coding which allows researchers to be an active reader while writing memos on particularly interesting codes (Strauss and Corbin, 1998; Charmaz, 2014). Memos are written records of the researchers thought process and will be taken throughout the entire study (Birks and Mills, 2015). They are essential to the discovery of the theory as they keep track of and explain the thought process of the researcher during the coding process (e.g., why the data was coded in a certain way; Birks and Mills, 2015). In order to establish consensus on the proper use of a code, pairs of coders will meet up to compare their individual codings and discuss potential discrepancies.

In the second stage we will use axial coding in order to identify links and relationships between the concepts and in order to create main and subcategories of the codes (Pandit, 1996). Explanatory and conceptual patterns and relationships are identified by looking for recurring phenomena, incidents, actions and interactions and by putting them in either main or subcategories (Strauss and Corbin, 1998). For example, all violence related codes could be categorized with the main code *violence* while subcategories could be named *behavioral violence* and *verbal violence*. In order to achieve consensus also on the more abstract categories or concepts, pairs of coders will once more meet to discuss their reasoning and approach of categorization. The emergence of new categories or changes in categories as well as their potential relation to existing literature (Locke, 2001) will be documented in the coding dictionary. Constantly comparing the data and the codes during the coding process ensures that the codes are congruent (Strauss and Corbin, 1998). In order to further explain the relationships between the concepts, we will start writing a story line, “a strategy for facilitating integration, construction, formulation and presentation of research findings through the production of a coherent grounded theory” (Birks and Mills, 2015, p. 176).

The final stage is the one of theoretical integration. In this stage a core category will be identified and the theory will be consolidated (Corbin and Strauss, 2008). A core category is defined by its ability to include all codes, sub and main categories, tying everything together to discover the theory (Corbin and Strauss, 2008). In this integrative process the storyline is developed further and results in a theory that is grounded in the data. In this process it is important to not have any preconceptions about the results and to look at the data objectively (Corbin and Strauss, 2008). Also in this final stage, pairs of coders will aim to reach consensus and will document all core categories and identified links in the coding dictionary.

### Ethics Statement

This study (ECP-164_14_03_2016) has been approved by the Ethical Review Committee Psychology and Neuroscience (ERCPN) of the Faculty of Psychology and Neuroscience of Maastricht University, The Netherlands. The review was done according to Dutch law and also in the light of the highest ethical standards in the Dutch, Anglo-American and European (Union) context.

## Anticipated Results

As very little is known about situational triggers of dark triad states at work, we can anticipate results only based on research on influencing factors of dark triad traits (Jonason et al., 2016) and maladaptive behaviors that are clinically relevant (i.e., schema modes; Keulen-de Vos et al., 2014). Research suggests for example that antisocial behavior at work (Robinson and O’Leary-Kelly, 1998; Lubit, 2002), competitive environments (Greenberg, 1990) and environments that can reduce a sense of behavioral accountability (e.g., cyberspace; Nevin, 2015) could facilitate dark personality expressions at work via social learning processes. In addition, scholars suggested that dark characteristics are more likely to emerge under periods of stress because this leads to a lack of cognitive resources that are needed to inhibit these dark impulses and motives in order to fulfill social expectations (Hogan and Hogan, 2001). Furthermore, research hints to the detrimental effects of traumatic experiences at work, such as victimization, threat, manipulation, bullying, and destructive leadership, which may potentially trigger expressions of dark personality states (e.g., Jonason et al., 2012; Sharf et al., 2014; Cheang and Appelbaum, 2015; Nevin, 2015; Smith et al., 2016). This is in line with research on cluster B personality disorders (e.g., antisocial personality disorder), showing that violent and delinquent behavior can be explained by an unfolding sequence of schema modes, feelings of vulnerability and abandonment or loneliness, which then lead to violent psychopathic behaviors, such as bullying and manipulation (Keulen-de Vos et al., 2014).

In sum, we expect to derive a taxonomy of triggers that represents an initial first step for conducting further quantitative research on these dynamics. For example, based on our taxonomy scholars could conduct field studies (e.g., diary studies with a cross-sectional or lagged design) as well as laboratory experiments to verify the factor structure of the triggers that we hope to identify as well as to test their causal impact on dark personality states. Further, we also call for additional qualitative studies on this topic in order to test if our taxonomy can be identified with other samples or data sources as well (i.e., proving consistency of our findings; Leung, 2015).

### Limitations

Although our study has a number of strengths, it also comes with several limitations and challenges. Firstly, grounded theory remains to some extent a subjective process bearing the risk of confirmation bias (Leung, 2015). Although subjectivity is considered an undesirable confounder in quantitative research, it is considered essential and even treasurable in qualitative research as it enriches the content of the findings (Leung, 2015). However, to make our findings more valid and generalizable (Johnson, 1997), we have interviewers/coders train their interviewing and coding skills and conduct pilot interviews, try to ensure that they are aware of the preconceptions they bring to the data coding process (Strauss and Corbin, 1998) and make our philosophical stance as transparent as possible. Importantly, the generalizability of grounded theory is partially achieved through the process of abstraction applied in the entire research process via the creation of codes, categories, and core categories (Strauss and Corbin, 1998).

Secondly, a major challenge concerns the attainment of high quality responses from participants in our interviews. As the interview topic touches the personal experiences of individuals and may also potentially explore deviant or illegal activities, it is of major importance to ensure that participants feel comfortable to report about this sensitive topic and to explicitly ensure anonymity and confidentiality (Larossa et al., 1981). We aim to build in *a priori* strategies for evaluating and terminating an interview should participants become severely distressed (Lee and Renzetti, 1990). These may include calling participants several days after the interview or providing them with a list of local counselors should the need arise (Corbin and Morse, 2003). In any case, we will try to make very explicit when approaching participants that speaking about this topic in the interview will not have any therapeutic implications and does not compensate seeking professional help to deal with stress or trauma.

Finally, as interviews will be conducted in German and English it is possible that cross-lingual and cross-cultural matters may arise, for instance data or categories derived in one language may not match the information derived in the other language (Squires, 2009). Although we will follow the recommendations of several authors with regard to a proper translation procedure (Temple, 2002; Van Nes et al., 2010) it is nonetheless important to acknowledge the cultural contexts people are situated in. To ensure a high quality of the project interviewers will receive training prior to conducting the interviews so as to allow them to master their interviewing skills and to minimize personal biases (Anderson, 2010). This also includes competence in cultural awareness; in other words, to be mindful of their own culture and how this may shape the interview process (Fontes, 2008).

### Implications and Conclusion

The results of our study will be of theoretical as well as of practical relevance. On a theoretical level, our study will add to research on the dark triad at work which has ignored more malleable conceptualizations of personality characteristics (i.e., personality states) until now. By investigating personality dynamics as they have occurred in a specific situation, we provide a more proximal and fine-grained perspective on dark triad expressions and their eliciting factors. Further, as research on the effect of work characteristics on personality states is still in its infancy, shedding light on *dark* personality dynamics at work broadens the domain of triggers and personality states that are of importance in organizational settings.

Identifying the impact of situational characteristics at work on individuals’ dark personality expressions is also highly relevant on a practical level. Research has shown that dark triad traits significantly relate to workplace deviance and counterproductive work behaviors (CWBs), such as workplace aggression, theft, and absenteeism (O’Boyle et al., 2012). These unethical organizational behaviors cause extreme damage to organizations. The Association of Certified Fraud Examiners estimated that globally, businesses suffer annual losses of U$2.9 trillion as a result of fraudulent activity (Moore et al., 2012). Building a categorization of dark triad triggers at work that will help organizations to design interventions that can prevent people from expressing their dark impulses (e.g., through appropriate job design or placement decisions) is therefore of great (economic) value. With our work, we want to support organizations to help their employees work on their dark side, thereby significantly improving people’s lives in the long run.

## Author Contributions

All authors listed have made substantial, direct and intellectual contributions to the work, and approved it for publication. AN came up with the idea for this project, contributed to and managed all steps of the design and writing process and took the lead in writing the protocol. JB, NB, RD, HE, EAJ, and GP contributed equally to this protocol in the areas of study design and support in manuscript writing. All co-authors are listed in alphabetical order.

## Conflict of Interest Statement

The authors declare that the research was conducted in the absence of any commercial or financial relationships that could be construed as a potential conflict of interest.
